# Ferroptosis and Cancer: Mitochondria Meet the “Iron Maiden” Cell Death

**DOI:** 10.3390/cells9061505

**Published:** 2020-06-20

**Authors:** Anna Martina Battaglia, Roberta Chirillo, Ilenia Aversa, Alessandro Sacco, Francesco Costanzo, Flavia Biamonte

**Affiliations:** 1 Department of Experimental and Clinical Medicine, “Magna Graecia” University of Catanzaro, 88100 Catanzaro, Italy; annamartinabattaglia@gmail.com (A.M.B.); roberta.chirillo@unicz.it (R.C.); ilenia.aversa@unicz.it (I.A.); alessandro.sacco@studenti.unicz.it (A.S.); fsc@unicz.it (F.C.); 2 Center of Interdepartmental Services (CIS), “Magna Graecia” University of Catanzaro, 88100 Catanzaro, Italy; 3 Research Centre of Biochemistry and advanced Molecular Biology, “Magna Graecia” University of Catanzaro, 88100 Catanzaro, Italy

**Keywords:** mitochondria, ferroptosis, cancer, cell death, iron, ROS

## Abstract

Ferroptosis is a new type of oxidative regulated cell death (RCD) driven by iron-dependent lipid peroxidation. As major sites of iron utilization and master regulators of oxidative metabolism, mitochondria are the main source of reactive oxygen species (ROS) and, thus, play a role in this type of RCD. Ferroptosis is, indeed, associated with severe damage in mitochondrial morphology, bioenergetics, and metabolism. Furthermore, dysregulation of mitochondrial metabolism is considered a biochemical feature of neurodegenerative diseases linked to ferroptosis. Whether mitochondrial dysfunction can, per se, initiate ferroptosis and whether mitochondrial function in ferroptosis is context-dependent are still under debate. Cancer cells accumulate high levels of iron and ROS to promote their metabolic activity and growth. Of note, cancer cell metabolic rewiring is often associated with acquired sensitivity to ferroptosis. This strongly suggests that ferroptosis may act as an adaptive response to metabolic imbalance and, thus, may constitute a new promising way to eradicate malignant cells. Here, we review the current literature on the role of mitochondria in ferroptosis, and we discuss opportunities to potentially use mitochondria-mediated ferroptosis as a new strategy for cancer therapy.

## 1. Introduction

Ferroptosis is a non-apoptotic, iron-dependent form of regulated cell death (RCD) occurring when the intracellular levels of lipid reactive oxygen species (L-ROS) exceed the antioxidant activity of glutathione-dependent peroxidase (GPX4) thus leading to the collapse of cellular redox homeostasis [1]. Ferroptosis is defined by three essential hallmarks: (i) oxidation of polyunsaturated fatty acid (PUFA)-containing membrane phospholipids; (ii) availability of redox-active iron; and (iii) loss of lipid hydroperoxide (LOOH) repair capacity [2]. The physiological function of ferroptosis as well as its involvement in multiple human diseases, such as ischemic organ injury, neurodegeneration, and cancer, have been established [3,4,5,6].

Unlike other RCDs, ferroptosis appears more like a cellular “sabotage” than a pro-active “suicide” mechanism [7]. While the “suicide” pathway (i.e., apoptosis, necroptosis, and pyroptosis) is actively triggered by a dedicated pro-death molecular machinery, the “sabotage” mechanism occurs when either inactivation or hyper activation of physiological processes causes a lethal metabolic imbalance with a so far undefined involvement of dedicated pro-death proteins [8]. During ferroptosis, cells are “sabotaged” by their own ongoing metabolism [9]. In cancer, such metabolic imbalance fosters the removal of tumor cells, thus suggesting ferroptosis is a sort of adaptive response which exerts a tumor suppressive function [10]. In this perspective, modulation of ferroptosis might represent a potential therapeutic approach for the so-called “persister” cancer cells, resistant to either standard chemotherapy or molecular-targeted therapies [11].

The essential role of cellular metabolism in ferroptosis is currently widely investigated. Mounting experimental evidence has demonstrated that numerous metabolic pathways, including cellular respiration (i.e., mitochondrial tricarboxylic acid (TCA) cycle and electron transport chain (ETC)), lipid metabolism, and amino acid metabolism contribute to ferroptosis through the generation of L-ROS [12,13]. Of note, iron metabolism may also induce ferroptosis through the lipid peroxide-generating Fenton Reaction [14].

As master regulators of oxidative phosphorylation (OXPHOS), mitochondria are the main intracellular producers of ROS [15]. Mitochondria are also focal hubs in iron metabolism and homeostasis [16]. The assessment of mitochondrial iron by using mitochondrion-selective fluorescent iron indicators or by using electron paramagnetic resonance revealed that, depending on the cell type, these organelles contain up to 20–50% of the total intracellular iron [17,18]. Mitochondrial iron mainly participates in iron–sulfur (Fe–S) cluster biogenesis and heme synthesis [19]; however, there also exists a free and redox active iron pool [20] which actively participates in the accumulation of mitochondrial ROS (mitoROS) [21]. In cancer cells, mitoROS act as second messengers in oncogenic signal transduction cascades, including those driven by mitogen-activated protein kinase (MAPK) and by the transcription factor NF-kB [22]. Upon accumulation, mitoROS can react with PUFAs in mitochondrial membranes leading to lipid peroxidation, mitochondrial DNA (mtDNA) damage, and subsequent defects in mtDNA-encoded subunits of the ETC complexes [23]. Such modifications have been observed not only in cancer cells but also in those diseases in which oxidative stress is increased such as chronic inflammations and neurodegenerative diseases [24,25].

All these observations are consistent with the potential involvement of mitochondria in ferroptosis. A series of molecular, pharmacological, and metabolomic analyses highlight that the metabolic activity of mitochondria, including both TCA cycle and ETC, is required for the generation of sufficient L-ROS to initiate ferroptosis [26]. Indeed, pharmacological induction of ferroptosis leads to mitoROS accumulation, mitochondrial fragmentation, alteration of the mitochondrial membrane potential (ΔΨ_m_), and ATP depletion [27,28,29]. Recent studies have also shown that a dysregulation of mitochondrial iron is typical of some neurological diseases, including Alzheimer’s disease, Huntington’s disease, Friedreich’s ataxia, and Parkinson’s disease which are all linked to ferroptosis [30,31,32,33].

Despite the involvement of mitochondria in ferroptosis being clearly defined, a comprehensive characterization of the underlying molecular mechanisms is still missing. Moreover, whether the involvement of mitochondria in ferroptosis is context-dependent or rather a general phenomenon is still unclear.

In this review, we summarize recent advances in our understanding of mitochondrial involvement in ferroptosis, and we discuss the potential opportunity to use mitochondria-mediated ferroptosis as a new strategy for cancer therapy.

## 2. Ferroptosis 

### 2.1. The Hallmarks of Ferroptosis

First described by Dixon et al. [34] in 2012, ferroptosis has been occasionally recognized as a unique form of cell death triggered by treatment with a panel of small molecules (i.e., erastin and rat sarcoma viral oncogene homolog (RAS)-selective lethal 3, RSL3) specifically selected to target mammalian tumor cells overexpressing mutant *RAS* oncogene.

Ferroptosis is an oxidative type of RCD morphologically, biochemically, and genetically distinct from apoptosis, necrosis, and other forms of non-apoptotic cell death [9,35]. 

Morphologically, ferroptotic cells exhibit ultrastructural changes in mitochondria such as volume reduction, increased bilayer membrane density, outer mitochondrial membrane (OMM) disruption, and disappearance of the mitochondrial cristae [34,36]. Furthermore, ballooning phenotype (i.e., the formation of a clear, rounded cell consisting mainly of empty cytosol) can be used to microscopically recognize ferroptotic cells [37]. Unlike apoptosis or necrosis, ferroptosis does not display formation of apoptotic bodies, cell shrinkage and chromatin condensation, or swelling of the cytoplasm and organelles and rupture of the cell membrane [38]. Unlike autophagy, ferroptosis does not display the formation of classical autophagosomes [38,39]. 

Biochemically, cells undergoing ferroptosis exhibit detrimental peroxidation of PUFAs in membrane phospholipids (PL-PUFAs) due to the increased intracellular amounts of redox active divalent iron (Fe^2+^) [40]. Normally, this process is carefully controlled by GPX4 which converts LOOH into the corresponding lipid alcohol [41]. The activity of GPX4 is closely dependent on glutathione (GSH) which, in turn, is synthetized from cysteine and glutamate, which have intracellular concentrations fine-tuned by the amino acid antiporter system *x*_c_^−^. System *x*_c_^−^ is a heterodimer composed of the 4F2 heavy subunit and the xCT light subunit, encoded by *SLC3A2* and *SLC7A11* genes, respectively. The light subunit xCT mediates the ATP-dependent exchange of extracellular cystine and intracellular glutamate across the cellular plasma membrane [42,43,44].

Genetically, ferroptosis is driven by several genes related to iron metabolism (transferrin receptor, *TFR1;* ferritin heavy chain, *FtH*; iron response element binding protein 2, *IREB2*), lipid synthesis (acyl-CoA synthetase family member 2, *ACSF2*; acyl-CoA synthetase long-chain family member 4, *ACSL4*) and oxidative stress pathways (ATP synthase F0 complex subunit C3, *ATP5G3*; citrate synthase, *CS*) [45,46].

**Figure 1:**
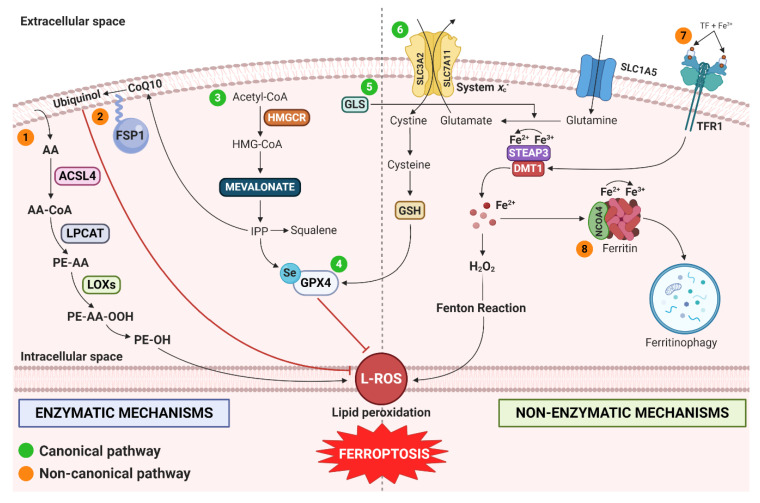
Regulatory metabolic pathways of ferroptosis. Metabolic pathways are divided into enzymatic (left panel) and non-enzymatic (right panel) mechanisms as well as into canonical (green dots) and non-canonical (orange dots). (1) Lipid metabolism pathway: AA and other PUFAs derived from lipid bilayers are metabolized by ACSL4 and LPCAT and then oxidized by LOXs to produce L-ROS. (2) FSP1-CoQ10-NADPH pathway: in plasma membrane, FSP1 reduces CoQ10 to ubiquinol which, in turn, blocks lipid peroxidation. (3) Mevalonate pathway: acetyl-CoA is converted to HMG-CoA by HMGCR. HMG-CoA is reduced to mevalonate which, in turn, is converted to IPP. As a result, a selenocysteine residue is added to catalytic center of GPX4. This event leads to GPX4 activation and ferroptosis inhibition. IPP also generates CoQ10, thus entering into the FSP1 pathway. (4) GPX4 pathway: GPX4 catalyzes the reduction of lipid peroxides thus preventing ferroptosis. (5) Glutaminolysis pathway: Extracellular glutamine, internalized through SLC1A5, is converted to glutamate by GLS. (6) Cystine deprivation-induced (CDI) ferroptosis pathways: amino acid antiporter system *x*_c_^−^ (composed by SLC3A2 and SLC7A11 subunits) mediates the exchange of extracellular cystine and intracellular glutamate. Cystine is converted in cysteine which, in turn, contributes to GSH production. Cystine deprivation triggers ferroptosis through GSH depletion. (7) Iron metabolism pathway: Fe^3+^-loaded TF is imported through TFR1. Fe^3+^ is converted in Fe^2+^ by STEAP3 and released into cytoplasm via DMT1. Fe^2+^ participates in Fenton Reaction, producing L-ROS and causing ferroptosis. (8) Ferritinophagy: Ferritin stores iron and reduces Fe^2+^ in Fe^3+^, limiting the Fenton Reaction. The NCOA4 binds ferritin mediating its autophagic degradation in a process called ferritinophagy. This mechanism promotes ferroptosis. *Abbreviation used*: AA, arachidonic acid; PUFAs, polyunsaturated fatty acids; ACSL4, long-chain-fatty-acid—CoA ligase 4; LPCAT, lyso-phosphatidylcholine acyltransferase; LOXs, lipoxygenase; L-ROS, lipid reactive oxygen species, PE, phosphatidylethanolamine; FSP1, ferroptosis-suppressor-protein 1 (also known as AIFM2); CoQ10, coenzyme Q10 (also known as ubiquinone); CoA, coenzyme A; HMGCR, 3-Hydroxy-3-Methylglutaryl-CoA Reductase; IPP, isopentenyl pyrophosphate; GPX4, glutathione peroxidase 4; SLC1A5, Solute Carrier Family 1 Member 5; GLS, glutaminase; CDI, Cystine deprivation-induced; SLC3A2, Solute Carrier Family 3 Member 2; SLC7A11, Solute Carrier Family 7 Member 11; GSH, glutathione; TF, transferrin; TFR1, transferrin receptor; STEAP3, STEAP3 Metalloreductase; DMT1, divalent metal transporter 1; L-ROS, lipid reactive oxygen species; NCOA4, nuclear receptor coactivator 4.

Ferroptosis is triggered by an imbalance between LOOH detoxification and iron-dependent L-ROS accumulation [1]. The metabolic pathways pushing the balance in favor of LOOH are graphically summarized in Figure 1.

The canonical pathway induces ferroptosis by directly inhibiting GPX4 or by causing GSH depletion [47]. Compounds such as RSL3 and others directly inhibit GPX4 thereby triggering ferroptosis without altering intracellular GSH levels [2,13]. Erastin and its derivatives are, instead, potent inhibitors of system *x*_c_^−^–mediated cystine import, thus affecting GSH bioavailability [48,49]. Glutamate (Glu) and glutamine (Gln) are also important regulators of ferroptosis. Elevated extracellular Glu levels can prevent the glutamate/cystine exchange, block system *x*_c_^−^ and trigger ferroptosis [50]. The mevalonate pathway leads to the production of coenzyme Q10 (CoQ10) and isopentenyl pyrophosphate (IPP), thus inhibiting ferroptosis [51,52]. 

The non-canonical pathway induces ferroptosis by increasing the intracellular labile iron pool (LIP) [49]. Although the knowledge about role of iron in ferroptosis requires clarifications, it is clear that a sufficient intracellular free Fe^2+^ amount is necessary in all mechanisms leading to the formation of LOOH, i.e., non-enzymatically lipid autoxidation and enzymatically lipid peroxidation [14]. Lipids can undergo spontaneous peroxidation in the presence of hydroxyl radicals (HO∙) generated from Fenton Reaction of redox active Fe^2+^ and hydrogen peroxide (H_2_O_2_) [53]. Alternatively, Fe^2+^ can serve as a cofactor for lipoxygenase (LOX) to enzymatically catalyze PUFA peroxidation [54]. 

Degradation of cytosolic ferritin through ferritinophagy is a key event in ferroptosis [55]. Ferritinophagy is mediated by the cargo receptor nuclear receptor coactivator-4 (NCOA4) which binds to and transports ferritin from cytosol to lysosomes leading to the release of free Fe^2+^ and to the consequent generation of lysosomal ROS [56].

The FSP1-CoQ10-NADPH pathway represents an independent parallel system which cooperates with GPX4 and GSH to suppress phospholipid peroxidation and ferroptosis [57]. Ferroptosis suppressor protein 1 (FSP1), formerly called apoptosis-inducing factor mitochondria associated 2 (AIFM2), exerts a protective effect on *GPX4* deletion-induced ferroptosis [57]. Myristylation of FSP1 leads to the recruitment of this protein to the plasma membrane where it reduces CoQ10 (also known as ubiquinone-10) to ubiquinol which, in turn, acts as a lipophilic radical-trap [58]. In both cases, FSP1 protects the cell by countering lipid peroxidation. Accordingly, *FSP1* knockout cell lines are significantly more sensitive to ferroptosis while *FSP1* overexpression can rescue cells from this type of cell death [38,57].

Interestingly, overloading cells with iron by using hemin, hemoglobin or iron chloride is per se sufficient to induce ferroptosis in some cell types [59]. Iron chelators, such as deferoxamine (DFO) or a variety of lipophilic antioxidants (i.e., vitamin E, ferrostatin-1 (Fer-1), and liproxstatin-1 (Lip-1)), potently inhibit ferroptosis by preventing the propagation of oxidative damage within the membrane [60,61].

### 2.2. The Role of Iron Metabolism in Ferroptosis

**Figure 2:**
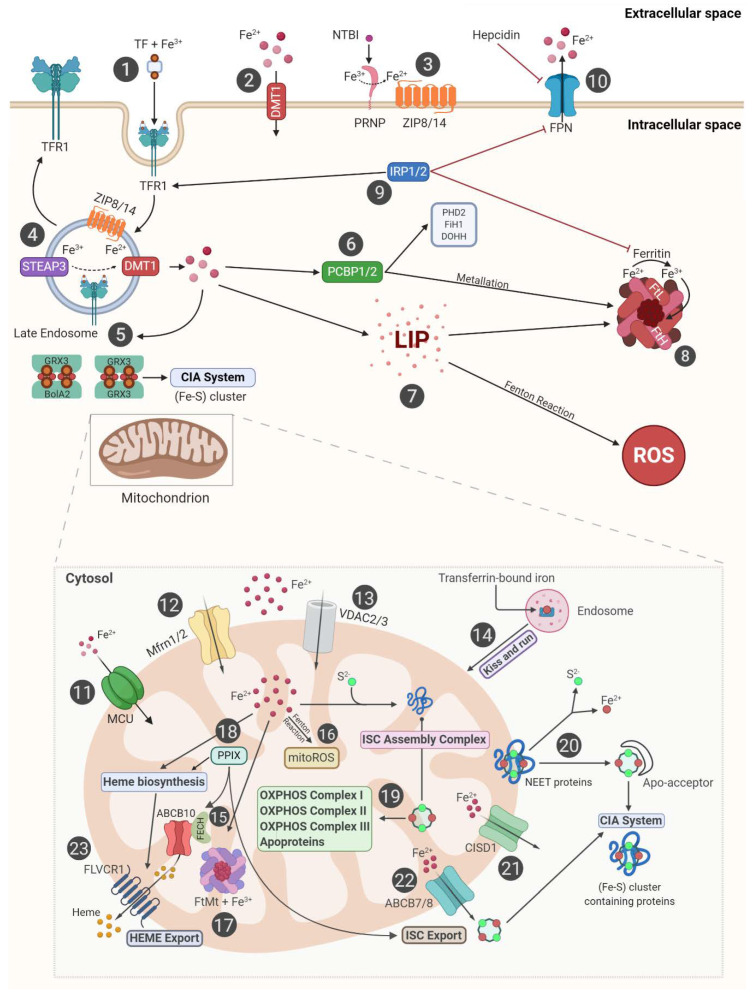
Iron crossroads from cytosol to mitochondria. Cytosolic iron metabolism: (1) TFR1 internalizes Fe^3+^-loaded TF through an endocytosis-mediated mechanism. (2) Fe^2+^ uptake is carried out by the transmembrane permeable channel DMT1. (3) NTBI enters cytoplasm through the zinc transporter ZIP 8/14 upon its reduction in Fe^2+^ mediated by PRNP. (4) Fe^3+^-loaded TF and NTBI are released in the endosome by TFR1 and ZIP8/14, respectively. STEAP3 converts Fe^3+^ to Fe^2+^ which, in turn, enters the cytoplasm via DMT1. After internalization, all these carriers are recycled to the cell surface. (5) GRX3 and BOLA2 constitute a heterotrimeric complex involved in the CIA system for (Fe–S) cluster formation. (6) PCBP1/2 iron chaperones bind iron and deliver it via direct protein–protein interaction with PHD2, FIH1, DOHH, and ferritin, in a process known as metallation. (7) LIP is a pool of free and redox-active iron which promotes ROS generation through a Fenton Reaction. (8) Ferritin is an iron-storage protein with ferroxidase activity, able to convert toxic Fe^2+^ in non-toxic Fe^3+^, thus preventing a Fenton Reaction. (9) IRPs coordinate iron homeostasis at the post-transcriptional level. IRP1/2 blocks degradation of *TFR1* mRNA and inhibits the translation of both ferritin subunits, *FtH* and *FtL*, and *FPN*. (10) FPN exports iron in the extracellular space; its activity is decreased by hepcidin that directly binds to FPN. Mitochondrial iron metabolism: (11) LIP released by lysosomes is rapidly taken up by MCU and internalized into mitochondria. (12) Mfrn1/2 imports Fe^2+^ from the intermembrane space of the mitochondria to the mitochondrial matrix. (13) VDAC2/3 mediates iron mitochondrial uptake. (14) Endosomal iron is delivered in mitochondria through the so-called “kiss and run” mechanism. (15) FECH forms an oligomeric complex with ABCB10 to synergistically promote mitochondrial iron import. (16) Fe^2+^ participates to Fenton Reaction-generating mitoROS. (17) FtMt, an H-type ferritin, is involved in mitochondrial iron storage. (18) PPIX incorporates iron to generate heme and mediates ISC export. (19) Mitochondrial iron can even enter the ISC assembly machinery, responsible for the maturation of all cellular (Fe–S) clusters; then, it can be mobilized to OXPHOS complex I/II/III. (20) NEET iron–sulfur proteins transfer their 2Fe–2S clusters to an apo-acceptor protein and CIA system. (21) CISD1, also called mitoNEET, regulates mitochondrial iron export. (22) ABCB7/8 are mitochondrial Fe–S cluster export proteins. (23) FLVCR1 mediates mitochondrial heme export. Abbreviations used: TFR1, transferrin receptor; TF, transferrin; DMT1, divalent metal transporter 1; NTBI, non-transferrin bound iron; ZIP 8/14, zinc finger iron proteins 8/14; PRNP, prion protein; STEAP3, six-transmembrane epithelial antigen of prostate 3; GRX3, glutathione-dependent oxidoreductase; BOLA2, BolA family member 2; CIA, cytosolic iron–sulfur cluster assembly; PCBP1/2, poly(RC) binding protein 1/2; PHD2, prolyl hydroxylase domain-containing protein 2; FIH1, factor inhibiting HIF-1; DOHH, deoxyhypusine hydroxylase; LIP, labile iron pool; ROS, reactive oxygen species; IRP, iron-responsive element-binding proteins; FtH, ferritin heavy chain; FtL, ferritin light chain; FPN, ferroportin; MCU, mitochondrial calcium uniporter; Mfrn1/2, mitoferrin 1/2; VDAC2/3, voltage-dependent anion-selective channel 2/3; FECH, ferrochelatase; ABCB10/7/8, ATP-binding cassette transporter 10/7/8; mitoROS, mitochondrial reactive oxygen species; FtMt, mitochondrial ferritin; PPIX, protoporphyrin IX; ISC, iron–sulfur (Fe–S) clusters; OXPHOS, oxidative phosphorylation; NEET proteins, also known as CDGSH iron sulfur domain 3; CISD1, CDGSH iron sulfur domain 1; ABCB7/8, ATP binding cassette subfamily b member 7/8; FLVCR1, feline leukemia virus subgroup C cellular receptor 1.

Given its unique redox properties, iron is often incorporated as a prosthetic group in enzymes and structural proteins and participates in many enzymatic reactions, thus representing a key player in many cellular biological processes [16]. The same features make iron potentially dangerous, as it can donate electrons to O_2_ and H_2_O_2_ to generate potentially harmful ROS such as hydroxyl radicals, hydroperoxyl radicals, and superoxide anions [53]. To ensure both fulfillment of metabolic needs and minimization of toxicity, cells are provided a complex protein network that tightly regulates iron import, storage, and detoxification (Figure 2) [62].

Briefly, transferrin (TF) imports circulating iron (Fe^3+^) into the cell by binding to its specific receptor TFR1 [63]. Once internalized, the TF–Fe^3+^–TFR1 complex localizes into the endosomes; here, in response to acidic conditions, iron is reduced to Fe^2+^ by the six-transmembrane epithelial antigen of the prostate 3 (STEAP3) and then exported into cytosol by divalent metal transporter 1 (DMT1) [64]. The majority of cytoplasmic iron is stored within ferritin, a nanocage composed of 24 subunits of both light (ferritin light chain, FtL) and heavy (ferritin heavy chain, FtH) types [65,66]. Ferritin heavy chain, in particular, is provided with ferroxidase activity through which it maintains iron in its ferric Fe^3+^ non-toxic form [67,68]. A small pool of cytoplasmic free Fe^2+^, referred to as LIP, directly catalyzes free radical formation via Fenton Reaction [69]. Excess Fe^2+^ is then oxidized to Fe^3+^ and exported by ferroportin (FPN) [70]. 

The expression of *TFR1* and *FtH* is regulated by the interaction between the iron regulatory proteins (IRPs) and the iron-responsive element (IRE), a stem-loop structure located in the 3’ UTR of *FtH* mRNA and in the 5′ UTR of *TFR1* mRNA. In response to cellular iron demand IRE/IRP interaction promotes *TFR1* mRNA stability and inhibits *FtH* translation, thus modulating cellular iron uptake and storage [71].

Overexpression of both *TF* and *TFR1* sensitizes cells to ferroptosis by enhancing iron uptake [72]; on the contrary, silencing *TFR1* can inhibit erastin-induced ferroptosis. In this regard, it has been recently demonstrated that heat shock protein beta-1 (HSPB1) significantly inhibits ferroptosis by repressing *TRF1* expression and, thus, reducing intracellular iron concentrations [38]. These proteins are a family of highly conserved molecular chaperones that, once activated by environmental stress, promote cell resistance to different types of cell death—including ferroptosis [73]. Heat shock protein family A member 5 (HSPA5), an endoplasmic reticulum (ER)-sessile chaperone, binds and stabilizes GPX4, thus indirectly counteracting lipid peroxidation in ferroptosis [73,74]. 

Changes of ferritin expression levels affect ferroptosis by altering the intracellular free and redox active iron pool. Torii et al. [75] have demonstrated that *NCOA4* overexpression reinforces ferritin degradation and then drives ferroptosis, while *NCOA4* knockdown suppresses ferritin degradation and inhibits ferroptosis. Increased expression of ferritin restrains the expansion of LIP and limits ferroptosis [76,77]. Indeed, suppression of IRE-binding protein *IREB2*, through RNA interference, significantly increases the expression of FtL and FtH subunits, thereby limiting erastin-induced ferroptosis [46]. 

Taken together, these data clearly indicate that the imbalance of intracellular iron homeostasis in favor of iron overload is pivotal for the induction of ferroptosis. 

### 2.3. Ferroptosis and Cancer 

Acting as an adaptive mechanism to eliminate malignant cells, ferroptosis constitutes a new tumor suppressing pathway [35]. Although initially defined as a new form of cell death occurring in *RAS* mutant cancer cells, it is now clear that the *RAS* pathway is not the sole determinant of ferroptosis occurrence in tumor [78,79]. 

Biochemically, two central events, intracellular iron accumulation and lipid peroxidation, are required for ferroptosis fulfillment in cancer cells [80]. The metabolite-mediated ways for inducing ferroptosis include decreasing cystine uptake through the inhibition of system *x*_c_^−^ and targeting GPX4, therefore, increasing iron concentration and ROS [81,82].

The tumor suppressor p53 plays a role in both inhibition and promotion of ferroptosis, depending on the cellular context. It can induce ferroptosis by inhibiting the transcription of the *SLC7A11* gene encoding the substrate-specific subunit of system *x*_c_^−^ [2,82]. Repression of *SLC7A11,* blocks cystine uptake and suppresses GPX4 activity, thus rendering cancer cells prone to undergo ferroptosis upon oxidant insults [83]. In this regard, in vivo studies have demonstrated that, while acetylation-defective p53 mutant (TP53-3KR) fails to trigger cell senescence, apoptosis and cell-cycle arrest, it is still able to suppress tumorigenesis via ferroptosis [84]. On the other hand, p53 is provided with an anti-ferroptotic function related to its capacity to boost antioxidant defense. This activity is most likely mediated by the p53/p21 axis activation that, preserving GSH and other thiols, suppresses phospholipid oxidation [85]. These observations are in line with the ability of p53 to limit erastin-induced ferroptosis in colorectal cancer (CRC) cells [86].

Ferroptosis is also promoted by the activity of p53 involvement in mevalonate pathway which generates a series of metabolites, including squalene and ubiquinone, with potential anti-ferroptotic activity [87]. When metabolic stress conditions occur, p53 promotes the expression of ATP-binding cassette subfamily A member 1 (*ABCA1*) that, in turn, regulates cholesterol efflux from the plasma membrane to the endoplasmic reticulum, causing inhibition of sterol regulatory element binding protein 2 (SREBP2) [88]. Inactivation of SREBP2 alters the mevalonate pathway, preventing the production of squalene and ubiquinone [89]. 

The tumor suppressor p53 can also induce ferroptosis by activating lipoxygenase ALOX12 function. Briefly, the transcriptional repression of *SLC7A11* leads to ALOX12-dependent ferroptosis upon oxidative stress [90]. Other lipoxygenases, including ALOXE3 and ALOX15B, are essential for ferroptosis occurrence in cancer. A comprehensive study showed that erastin-induced ferroptosis is rescued by silencing either *ALOX15B* or *ALOXE3* in transformed fibroblasts (BJeLR) and fibrosarcoma (HT-1080) cells [42]. Epigenetic regulation also plays a key role in ferroptosis. Loss of function mutations of the tumor suppressor BRCA1-associated protein 1 (BAP1) has recently been linked to ferroptosis [91]. This BAP1, a nuclear-located deubiquitinase (DUB), promotes the formation of the polycomb-repressive–deubiquitinase (PR-DUB) complex and reduces histone 2A ubiquitination (H2Aub) on the *SLC7A11* promoter [92]. The consequent downregulation of *SLC7A11* blocks ferroptosis, as it leads to cystine starvation and depletion of GSH [93]. 

A plethora of long non-coding RNAs (lncRNAs) and microRNAs (miRNAs) have recently been reported to regulate ROS metabolism and ferroptosis [94,95]. For example, P53RRA lncRNA promotes cell-cycle arrest, apoptosis, and ferroptosis by binding to Ras GTPase-activating protein-binding protein 1 (G3BP1) and preventing its interaction with p53 which is consequently retained in the cell nucleus [96]. The microRNA 137 inhibits ferroptosis by targeting *SLC1A5* which results in dysfunction of the glutamine transporter in cancer cells. The metabolism of L-Gln contributes to the formation of oxidizable lipids to induce ferroptosis. The Gln importer SLC1A5/SLC38A1, glutaminases 2 (GLS2), and glutamic-oxaloacetic transaminase 1 (GOT1) are required for Gln uptake and metabolism to Glu and ultimately to α-ketoglutarate (α-KG). Accordingly, miR-137 overexpression suppresses erastin/RSL3-induced ferroptosis in melanoma cells [97]. 

To sum up, numerous genes/proteins and metabolic pathways are involved in the execution of ferroptosis in cancer cells. A more extensive list of the main molecules implicated in ferroptosis is reported in Table 1.

Morphologically, ferroptotic cancer cells exhibit alterations of mitochondrial morphology and cristae structure. Upon treatment with erastin in vitro, ferroptotic BJeLR cancer cells are usually rounded up and detached [45]. Transmission electron microscopy (TEM) reveals the presence of small mitochondria with increased mitochondrial membrane density and vanishing of mitochondrial cristae. The cell membrane remains intact and the nucleus shows a normal size, without chromatin concentration [98,99].

### 2.4. Ferroptosis Is a New Promising Target for Cancer Treatment

Metabolic reprogramming leads to the acquisition of ferroptosis sensitivity as part of an escape strategy against other therapies [112]. This observation strongly supports the potential use of ferroptosis initiating therapies (FITs) in the management of the so-called “persister cells”, a subset of cancer cells able to survive upon treatment with several rounds of chemotherapy drugs, leading to tumor relapse [113]. 

Several drugs targeting ferroptosis have been tested as a new approach in anti-tumor therapies in vitro. Overall, these drugs can be classified as follow: (i) drugs directly or indirectly inhibiting system *x*_c_^−^ (i.e., erastin, sorafenib, and sulfasalazine); (ii) drugs inhibiting GSH synthesis through the suppression of γ-glutamylcysteine synthetase (GCS) (i.e., buthionine sulfoximine, BSO); and (iii) drugs inhibiting GPX4 (i.e., RSL3, withaferin A and FIN56). 

Among the abovementioned drugs RSL3 and erastin, the two main ferroptosis inducers have been used in a variety of tumor models in vitro [114,115]. Both drugs, however, do not meet the pharmacokinetic standards for in vivo application and need to be further optimized for clinical application [116]. Sorafenib is an FDA-approved multi-kinase inhibitor for treatment of advanced renal cell carcinoma (RCC) and advanced HCC. Sulfasalazine (SSZ) disrupts iron metabolism through cystine uptake blockade, resulting in ferroptosis of glioma cells [117].

A drug screening analysis has indicated that some of the well-known chemotherapeutics, such as cisplatin, altretamine, and artesunate, are able to promote ferroptosis [118]. Cisplatin leads to GSH depletion and GPX4 inactivation [119]; indeed, it is emerging as inducer of both ferroptosis and apoptosis in A549 non-small cell lung cancer (NSCLC) cells and HCT116 CRC cells [119]. Altretamine (hexamethylmelamine), an FDA-approved alkylating antineoplastic drug used for treating ovarian cancer, inhibits GPX4 and effectively kills U-2932 diffuse large B cell lymphoma (DLBCL) cells in vitro [120]. Artesunate (ART) increases ROS generation in cancer cells, and its antitumor effect is carried out through ferroptosis in a variety of neoplastic diseases like pancreatic ductal adenocarcinoma (PDAC), epithelial ovarian cancer (EOC), and HNCs [39].

Recently, nanocarriers have been proposed as an efficient approach to induce ferroptosis in cancer cells in vitro and in vivo [121]. Doxorubicin, packed into mesoporous carbon nanoparticles, induces ferroptosis in breast cancer (MCF7) cells, in A549 cells, and in human cervical carcinoma (HeLa) cells [122]. The nano-targeting of withaferin A, a natural ferroptosis-inducing agent, efficiently kills high-risk neuroblastoma cell lines and suppresses growth of neuroblastoma xenografts in mice [123]. Iron-based nanoparticles can release Fe^2+^ and Fe^3+^ in acidic lysosomes, inducing ferroptosis, ultimately suppressing tumor growth [124]. In this regard, treatment with small (∼6 nm) surface-functionalized poly(ethylene glycol)-coated (PEGylated) silica nanoparticles (C’ dots) can induce ferroptosis in tumor xenografts by delivering iron into cells [125]. Since exogenous iron overload (e.g., ferric ammonium citrate) is not able to induce ferroptosis in all cell types, it is likely that treatment with C’ dots is effective in a cell-specific manner [2]. 

Numerous other molecules have been shown to trigger ferroptotic cell death in cancer cells and future studies will be necessary for their validation in real clinical settings.

The array of compounds able to induce ferroptosis in cancer cells is summarized in Table 2. Nonetheless, for a more detailed discussion about drugs currently used in ferroptosis-based cancer treatment, we recommend the exhaustive review by Bin Lu et al. [11].

## 3. Mitochondria at the Crossroad of Ferroptosis and Cancer Suppression

Mitochondria play a pivotal role in metabolic plasticity in malignant cells, as well as in the regulation of many RCD processes, and ferroptosis is no exception [140]. Mitochondria seem to be involved in ferroptosis induced by cystine deprivation (CDI) which, indeed, is associated with mitochondrial membrane hyperpolarization and lipid peroxide accumulation [26]. In agreement, erastin treatment boosts the production of mitoROS [26] which, in turn, cause opening of mitochondrial permeability transition pore (mPTP), dissipation of ΔΨ_m_ and ATP depletion [141]. Cells undergoing ferroptosis exhibit mitochondria fragmentation and specific changes in mitochondrial morphology such as reduction of mitochondrial cristae and decrease in mitochondrial size [142]. 

However, some questions remain controversial. Whether mitochondrial dysregulation is able, per se, to initiate this type of cell death or it is just a consequence of the metabolic imbalance is unclear. Based on Gaschler et al. [134], cells lacking mitochondria are still sensitive to ferroptosis. Conversely, according to Gao et al. [26], inhibition of TCA cycle and mitochondrial ETC can rescue cells from mitochondrial membrane hyperpolarization, lipid peroxide accumulation, and ferroptosis. Mitochondrial role in ferroptosis seems context dependent. Upon cystine deprivation, mitochondria contribute to reducing GSH and to promoting ROS production. Glutaminolysis is required for CDI ferroptosis.

Of note, in the absence of Gln, neither cystine starvation nor erastin inhibition of system *x*_c_^−^ can induce ferroptosis [26]. Mitochondrial free iron accumulation exacerbates erastin-mediated ferroptosis [143]. Alternatively, sequestering iron within mitochondria via overexpression of mitochondrial ferritin (FtMt) can counteract erastin-induced cell death, both in vitro and in vivo [76]. Supporting this last observation, impaired mitochondrial iron metabolism is a common feature of many neurodegenerative diseases (i.e., Alzheimer’s, Parkinson’s, Huntington’s diseases) [144,145,146,147], all linked to ferroptosis. Morphologically, mitochondria in brains isolated from mice models of these diseases exhibit disrupted cristae [148] that are reminiscent of those observed in ferroptosis. Upon GPX4 inhibition, ferroptosis appears, instead, independent of mitochondria [26].

**Figure 3:**
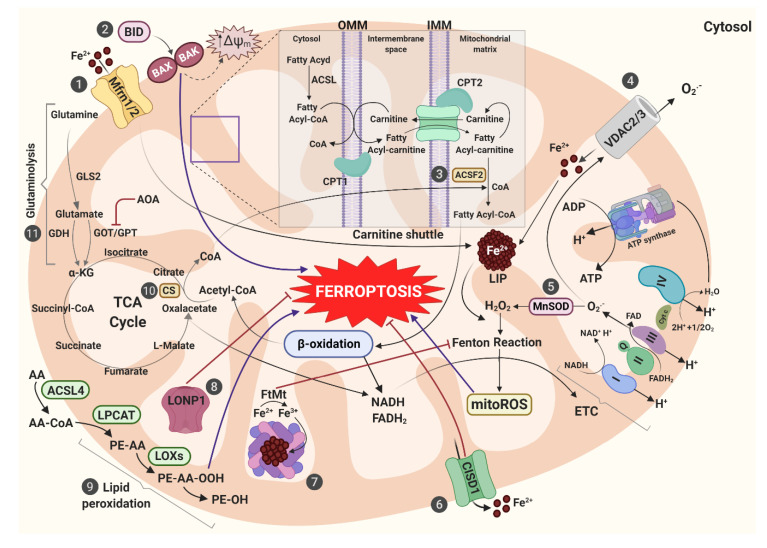
Mitochondrial metabolic processes in ferroptosis. (1) Iron uptake via Mfrn1/2 increases LIP amount, promoting mitoROS generation through Fenton Reaction. (2) BID triggers ferroptosis through BAX and BAK activation and the consequent dysregulation of ΔΨ_m_. (3) ACSF2 regulates activation of fatty acids derived from carnitine shuttle mechanism, providing the specific lipid precursor for β-oxidation. (4) VDAC2/3 imports Fe^2+^ into mitochondria. Fe^2+^ contributes to enhance LIP which, in turn, generates mitoROS. (5) MnSOD converts superoxide anion (O_2_^−^) from ETC to hydrogen peroxide (H_2_O_2_) which takes part into Fenton Reaction, thus promoting ferroptosis. (6) CISD1 regulates mitochondrial iron export acting as ferroptosis suppressor. (7) FtMt prevents Fenton Reaction through iron-storage and ferroxidase activities. (8) LONP1 maintains mitochondrial integrity, preventing ferroptosis induction. (9) ACSL4, LPCAT, and LOXs activate lipid peroxidation, driving ferroptosis. (10) CS regulates fatty acid synthesis through the release of CoA, a precursor for β-oxidation, thus inducing ferroptosis. (11) Glutamine is converted to glutamate by the mitochondrial isoform GLS2. Glutamate is converted in α-KG by GDH and GOT/GPT enzymes, thus providing fuel for TCA cycle and lipid biosynthesis. Abbreviations used: Mfrn1/2, mitoferrin 1/2; LIP, labile iron pool; mitoROS, mitochondrial reactive oxygen species; BID, BH3 interacting-domain death agonist; BAX, Bcl-2-associated X protein (also known as bcl-2-like protein 4); BAK, Bcl-2 homologous antagonist killer; ACSF2, acyl-CoA synthetase family member 2; VDAC2/3, voltage-dependent anion channels 2/3; MnSOD, mitochondrial superoxide dismutase; ETC, electron transport chain; CISD1, CDGSH Iron Sulfur Domain 1; FtMt, mitochondrial ferritin; LONP1, lon peptidase 1; ACSL4, long-chain-fatty-acid—CoA ligase 4; LPCAT, lyso-phosphatidylcholine acyltransferase; LOXs, lipoxygenase; AA, arachidonic acid; PE, phosphatidylethanolamine; CPT1/2, carnitine palmitoyltransferase 1/2;CS, citrate synthase; CoA, coenzyme A; GLS1/2, glutaminase 1/2; α-KG: alpha-ketoglutarate; GDH, glutamate dehydrogenase; GOT, glutamic oxaloacetic transaminase; GTP, glutamic pyruvic transaminase; TCA cycle (tricarboxylic acid cycle); AOA, amino-oxyacetic acid.

In the following sections, we review the morphological, metabolic, and energetic features that closely relate mitochondria to ferroptotic cell death (Figure 3).

### 3.1. Mitochondrial Morphological Features in Ferroptosis

The ultrastructural changes of mitochondria are considered the morphological trademark of ferroptosis that help to distinguish this new type of RCD from apoptosis, necroptosis, and autophagy [99]. These changes occur upon both pharmacological and genetical induction of ferroptosis in all cell types. Considering that biomarkers exclusively associated with ferroptosis are missing, the detection of typical mitochondrial morphological changes by TEM represents one of the few available methods for the identification of ferroptosis [98]. A list of the available methods for the in-depth characterization of mitochondrial function in ferroptosis is reported in Table 3.

The morphological features of ferroptotic cells can be classified based on the extent of mitochondria fragmentation and their distribution: (i) uniformly distributed, elongated mitochondria, (ii) uniformly distributed, fragmented mitochondria, (iii) fragmented mitochondria mainly distributed close to the nucleus, (iv) small rounded mitochondria located close to the nucleus [27,153,154]. As previously reported, shrinkage of mitochondria with enhanced mitochondrial membrane density, volume reduction, and vanishing of mitochondrial cristae have been observed in ferroptosis following erastin treatment in BJeLR cells [45]. Induction of ferroptosis by *GPX4* knockdown in immortalized fibroblasts and kidney tissue-derived cells has been associated with OMM rupture as observed using TEM. In mouse embryonic fibroblast (MEF) cells (Pfa1 cells), RSL3 treatment induces OMM rupture in a time-dependent manner [116]. 

In contrast, no morphological features related to necrosis (cytoplasmic swelling, plasma membrane rupture), apoptosis (chromatin condensation and apoptotic bodies) or autophagy (formation of double-membrane enclosed vesicles) were observed following erastin treatment in cancer cells [34,36]. 

### 3.2. Mitochondrial Energetic Metabolism in Ferroptosis

Mitochondrial metabolism and ferroptosis closely interact with one another. In cystine-deprivation conditions, mitochondrial metabolism significantly contributes to L-ROS generation and ferroptosis [26]. As such, mitochondrial damage and mitoROS production occur upon inhibition of xCT or upon cystine starvation, but are not required for ferroptosis induced by GPX4 inhibition [155].

Concerning the role of glutaminolysis, it has been reported that conditions of cystine deprivation promote mitochondrial respiration and the rapid depletion of GSH, thus inducing ROS accumulation, lipid peroxidation and ferroptosis. In the absence of Gln, neither cysteine starvation nor erastin inhibition of system *x*_c_^−^ can induce ferroptosis [26]. Glutaminases 1 (GLS1) and GLS2 catalyze the conversion of Gln into Glu [156]. Of note, ferroptosis can be prevented by pharmacological or the genetic inhibition of mitochondrial isoform GLS2, while the cytoplasmic isoform GLS1 is not able to block this type of RCD [50]. Moreover, *GLS2* is a transcriptional target of p53 and is up-regulated during p53-dependent ferroptosis [157]. Transaminases convert Glu into α-KG through the transamination process [158]. Both treatment with the transaminases inhibitor aminooxyacetic acid (AOA) and knockdown of the transaminase *GOT1* inhibit CDI ferroptosis in MEFs [34,158]. 

Blockade of glutaminolysis can be counteracted by supplying TCA cycle with metabolites such as αKG, succinate, fumarate, and malate. These intermediates, all downstream of glutaminolysis, can replace the role of Gln in L-ROS accumulation in both MEFs and HT-1080 cells [26], thus supporting the involvement of the TCA cycle in CDI ferroptosis.

Several enzymes of the TCA cycle (i.e., fumarate hydratase, FH, aconitase, ACO, and citrate synthase CS) are necessary for ferroptosis triggered by cystine starvation or by erastin treatment [26]. Accordingly, inhibition of the TCA cycle mitigates ΔΨ_m_ hyperpolarization, lipid peroxide accumulation, and ferroptosis [49]. The knockdown of dihydrolipoamide dehydrogenase (*DLD*), a component of α-KG dehydrogenase complex, blocks the increase of L-ROS amount and ΔΨ_m_ caused by cystine deprivation- or sulfasalazine treatment-induced ferroptosis in HNC [159]. Loss of FH, which also exerts a tumor suppressor function, confers resistance to CDI ferroptosis in renal cancer cells [26].

The TCA cycle supports electron transport activity of protein complexes located in the inner mitochondrial membrane (IMM). As shown by Gao et al. [26], inhibition of the ETC mitochondrial complex I, complex II, complex III, and complex IV suppresses L-ROS accumulation and ferroptosis induced by cystine starvation or erastin treatment in HT-1080 cells. As master regulator of OXPHOS, mitochondria are the major source of ROS [160]. Indeed, cells with disrupted glycolysis are vulnerable to ferroptosis by rewiring cell metabolism to OXPHOS [161]. 

Mitochondrial fatty acids metabolism represents an important source for lipid peroxides production during ferroptosis [155]. Increase of the proton conductance of the IMM, ETC inhibition and mPTP opening constitute three of the main mechanisms through which fatty-acid metabolism modulates mitochondrial energy to provoke lipid oxidation [45]. Both ACSF2 and CS regulate synthesis and activation of fatty acids: in detail, ACSF2 forms an activating thioester bond between the fatty acid and CoA, while CS catalyzes the first reaction of the TCA, condensing acetyl-CoA and oxaloacetate to form citrate [45]. Dixon et al. [34] demonstrate that *CS* and *ACSF2* knockdown, in both HT-1080 and BJeLR cells, blocks erastin-induced ferroptosis.

Overall, these results strongly support the role of mitochondrial metabolism in CDI ferroptosis and corroborate the hypothesis that ferroptosis acts as a tumor suppressive mechanism potentially useful for cancer therapeutic approaches.

### 3.3. Mitochondria and Iron Metabolism

Iron is the most prevalent metal inside the mitochondria and actively participates to the physiological functions of these organelles [162,163]. Once imported into the cell, iron can be delivered to mitochondria by several mechanisms including (i) the transient interaction between the transferrin-bound iron within endosomes and the OMM, the so-called “kiss and run” model; (ii) the uptake of low/high molecular weight iron complexes from the cytosolic LIP; and (iii) the transfer of iron bound to metallochaperones such as the poly(C)-binding proteins (PCBPs) (Figure 2) [164,165]. 

Since mitochondrial iron metabolism mainly occurs in the mitochondrial matrix, iron must cross both the OMM and IMM. Iron transport across the IMM is an active process dependent on the membrane transporter mitoferrin 1 (Mfrn1) and its homolog mitoferrin 2 (Mfrn2) (Figure 2). Dysregulation of Mfrn1/2 leads to mitochondrial iron accumulation and oxidative damage [166]. Of note, recent studies highlight that Mfrn1/2 is impaired in neurological diseases, such as Alzheimer’s disease, Huntington’s disease, Friedreich’s ataxia (FRDA), and Parkinson’s disease, which are all linked to ferroptosis [167].

The voltage-dependent anion channels (VDACs), located in the OMM, also regulate the influx of iron in mitochondria (Figure 2) [168]. Erastin treatment induces VDAC2/3 opening and is associated with mitochondrial iron accumulation and iron-dependent ferroptosis [169,170].

Following import, mitochondrial iron primarily acts as a cofactor in Fe–S cluster-containing proteins (i.e., NADH:ubiquinone oxidoreductase) and heme-containing proteins (i.e., cytochrome c, cytochrome c oxidase, and succinate dehydrogenase) all of which are components of the IMM complexes of the ETC [171]. The biogenesis of Fe–S cluster is driven by the activation of the mitochondrial protein frataxin (FXN), which functions as iron chaperone [172]. Friedreich’s ataxia (FRDA), caused by decreased expression of FXN, is characterized by mitochondrial iron accumulation, mitochondrial dysfunction and increased oxidative stress. Of note, ferroptosis inhibitors have been effectively tested as potential therapeutic approach on primary FRDA patient-derived fibroblasts [173]. Recently, Jing Du et al have demonstrated a link between FXN and ferroptosis in cancer. Suppression of FXN impairs mitochondrial morphology, prevents Fe–S cluster assembly and enhances CDI ferroptosis in HT-1080 cancer cells [173].

Mitochondria contain a labile iron pool which is extremely redox active [25]. In physiological conditions, free iron homeostasis is tightly controlled by FtMt [174]. FtMt is structurally similar to cytosolic FtH and has ferroxidase- and iron-binding activities similar to cytosolic ferritin [175]. Mitochondrial ferritin protects against mitochondrial ROS accumulation [176] that, otherwise, may injure proteins, lipids and DNA within the mitochondria and impair ATP production, causing energy stress [177]. Downregulation of FtMt enhances mitochondrial free iron accumulation and inevitably leads to mitoROS accumulation and ferroptosis [76].

The importance of the mitochondrial iron metabolism in ferroptosis is further supported by the role of the new discovered iron-sulfur proteins (2Fe-2S) NEET. These proteins mediate the export of sulfur ions and iron between the cytosol and the mitochondria [178]. Deletion of the mitochondrial isoform CDGSH iron sulfur domain 1 (CISD1), also known as mitoNEET, causes mitochondrial iron accumulation and generation of mitochondrial lipid peroxides contributing to ferroptosis [29]. Interestingly, *CISD1* knockdown mice exhibit many features of Parkinson’s disease [179].

### 3.4. Ferroptosis Mediated by Mitochondrial VDACs

Voltage-dependent anion channels (VDAC1, VDAC2, and VDAC3) operate at the OMM to control the trafficking of ions and metabolites between cytosol and mitochondria [168]. Consequently, loss of VDAC2/3 affects mitochondrial activity by disrupting ΔΨ_m_ homeostasis. In this regard, Yagoda et al. [28] have demonstrated that binds to and targets VDAC2/3 resulting in ΔΨ_m_ alteration and eventually ferroptosis in cancer cells harboring *RAS* mutations. In agreement, siRNA-mediated knockdown of *VDAC2/3* is able to attenuate erastin-induced ferroptosis [78]. In the same study, Yagoda et al. [28] have shown that erastin treatment also breaks down the expression of both VDAC2 and VDAC3. In melanoma cells, erastin induces the activation of the E3-ligase Nedd4 which, in turn, induces VDAC2/3 ubiquitination and ferroptosis [169]. Similarly, RSL3 treatment causes VDAC2/3 degradation through Nedd4 [26]. However, knockdown of *VDAC2/3* suppresses the sensitivity of cells to erastin but not to RSL3 [180].

Although VDACs have been largely considered constitutively open, recent studies show that VDACs conductance capacity is inhibited by intracellular free tubulin abundance [181]. In preclinical models of osteosarcoma, microtubule-destabilizing agents increase cytoplasmic free tubulin causing a decrease of ΔΨ_m_ [182]. Erastin and other analogues block the tubulin-dependent VDAC closure, thus leading to an increase of ΔΨ_m_ and ROS-dependent mitochondrial dysfunction, bioenergetic failure and, ultimately, ferroptosis in HepG2 and Huh7 human hepatocarcinoma cells [36].

### 3.5. Other Pathways

Erastin-induced ferroptosis is linked to mitochondrial transactivation of Bcl-2 family member BH3-interacting domain death agonist (BID) (Figure 3) [27]. This protein acts as a connection bridge between surface death receptors (e.g., Fas and tumor necrosis factor-α, TNF-α) and the core extrinsic apoptotic pathway in mitochondria [183]. Its activation is mediated by caspase-8 cleavage [184]; BID is then translocated into the mitochondria where it activates the pro-apoptotic proteins BAX and BAK [185]. Of note, knockout of *BID* in neural cells, using CRISPR/Cas9 approach, preserves mitochondrial integrity and function, and mediates a neuroprotective effect against ferroptosis [27]. The specific mechanisms of BID in paradigm of ferroptosis need further investigations.

Lon peptidase 1 (LONP1) mediates the selective degradation of misfolded or oxidatively damaged polypeptides in the mitochondrial matrix and maintain the integrity of the mitochondrial genome (Figure 3) [186]. In the PANC1 cell line, erastin-induced ferroptosis enhances the expression of mitochondrial *LONP1*. Conversely, *LONP1* inhibition leads to the activation of NRF2/KEAP1 signalling pathway and to the up-regulation of *GPX4*, thus inhibiting ferroptosis [187].

An overview of the crucial mitochondrial actors involved in ferroptosis induction are reported in Table 4.

## 4. Discussion

Ferroptosis occurs when lipid hydroperoxide detoxification mediated by GPX4 activity is reduced to such an extent that it becomes insufficient to restrain iron-dependent membrane PUFA oxidation and toxic ROS accumulation [2]. 

As a main source of cellular ROS, mitochondrial metabolism is likely to play a pivotal role in the execution of ferroptosis [26]. A survey of the literature clearly highlights that ferroptosis is accompanied by severe morphological and functional mitochondrial damages and that, at the same time, a proper function of mitochondrial bioenergetic metabolism is mandatory for the initiation and the accomplishment of this new type of cell death [35,45,99]. Interference of key regulators of mitochondrial lipid metabolism (i.e., ASCF2 and CS), glutamine metabolism (i.e., GLS2), TCA cycle (i.e., FH) and other signaling pathways consistently enhance sensitivity to ferroptosis [26,34]. Nonetheless, our knowledge of the molecular mechanisms underlying these events are still limited and additional studies are warranted.

Suggestive evidence of the ferroptosis/mitochondria crosstalk is represented by the strong iron dependency of this RCD [26,38]. Intracellular iron accumulation can generate ROS and cause oxidative stress via Fenton Reaction, thereby promoting lipid peroxidation [61]. Mitochondrial iron homeostasis is altered to satisfy the redox active iron demands for propagating ferroptosis [26,99]. A direct in vivo evidence for the involvement of mitochondrial iron metabolism in ferroptosis is represented by neurodegenerative diseases, whose pathogenetic mechanisms have been recently linked to ferroptosis [167]. Whether mitochondrial iron crosstalk with cytosolic iron or, otherwise, mitochondrial iron metabolism is independent, to a certain extent, from cytosolic iron metabolism is still under debate [16]. For instance, when heme synthesis is inhibited in the mitochondrion, iron continues to enter these organelles [192]. This finding may suggest the lack of communication between cytoplasm and mitochondrion, as iron continues to be transported into this organelle irrespective of heme synthesis inhibition. Otherwise, it can suggest that iron continues to enter the mitochondrion in an effort to rescue heme synthesis. A recent work by Li et al. [193], highlighted that fibroblasts and lymphoblasts from Friedreich’s ataxia (FA) patients display cytosolic iron-deficiency. Overexpression of mitochondrial ferritin (FtMt) in the mitochondrion leads to mitochondrial iron-loading and cytosolic iron deprivation [194]. Collectively, these data suggest that mitochondrial iron metabolism can mediate ferroptosis by modulating whole-cell iron processing.

Metabolic plasticity is a critical property that gives cancer cells the edge for expanding, persisting after therapeutic hits and evading immune surveillance [195]. Recently, metabolic reprogramming has been associated with acquired sensitivity to ferroptosis, thus opening up new opportunities to treat therapy-insensitive tumors [1]. Of note, either the genetic manipulation or the pharmacological targeting of proteins involved in ferroptosis have been found to induce cell death in a wide range of cancer cells [11]. The susceptibility of different types of cancer cells to ferroptosis is though significantly variable [155]. Based on some recent studies, the different sensitivity of cancer cells to ferroptosis depends on their basic metabolic status [78]. Considering the pivotal role of mitochondria in tumor cell metabolic rewiring, it is possible that modulation of the mitochondrial metabolic pathways might reshape the tumor microenvironment thus leading to ferroptosis-mediated tumor suppression. To make some examples, cancer stem cells frequently present a mitochondrial metabolic shift from glycolysis to OXPHOS [196], that can be exploited to make these cells vulnerable to ferroptosis. Glutaminolysis is used by the majority of cancer cells to satisfy their bioenergetic requirements [197]. Since its role in promoting ferroptosis, glutaminolysis may represent a nodal point of vulnerability for cancer cells and a potential target for novel anti-tumor strategies [198]. Iron addiction is a characteristic of cancer cells [199]. Modulation of both mitochondrial FXN and NEET proteins has been associated with CDI ferroptosis in cancer cells.

Overall, these findings provide a clear support for the potential use of mitochondria-mediated ferroptosis in cancer treatment. Future studies exploring the effects of mitochondrial metabolic rewiring in in vivo models of ferroptosis would be necessary to confirm the role of this cell death as new exciting frontier in cancer biology. 

## Figures and Tables

**Table 1:** Inducers and inhibitors of ferroptosis.

Molecule	Targets	Mechanisms	Effect	References
p53	*SLC7A11*	Inhibition of System *x*_c_^−^	Pro-ferroptosis	[2,83]
BAP1	*SLC7A11*	Inhibition of System *x*_c_^−^		[91]
IFNγ	*SLC7A11* *SLC3A2*	Induction of lipid peroxidation		[80]
EGFR	MAPK	Cystine deprivation		[100]
HO-1	Heme	Heme degradation: cellular iron availability		[101]
P53RRA lncRNA	G3BP1	p53 nucleus retention		[96]
FANCD2	GPX4 Iron metabolism genes	GPX4 inhibition and cellular iron availability		[102]
as-lncRNA SLC7A11	*SLC7A11*	Inhibition of System *x*_c_^−^		[103]
SAT1	lipoxygenases	Lipid peroxidation		[104]
DPP4	NOXs	Lipid peroxidation		[86]
ACSL4	fatty acids	Conversion of free fatty acids into fatty CoA ester		[105]
miR-155	ROS	Increase of ROS levels through inhibiting FOXO3a expression		[106]
miR-206	ROS	Increase of ROS production by targeting SOD1		[107]
HSPB1	actin dynamics	Cellular iron availability		[108]
**NRF2**	Iron metabolism genes *SLC7A11, HO-1, GPX4, G6PD*	Inhibition of System *x*_c_^−^, Cellular iron availability	Anti-ferroptosis	[109]
**miR-137**	*SLC1A5*	Accumulation of MDA		[97]
**miR-448-3p**	ROS	Reduces of NOX2-dependent ROS production		[110]
**miR-25**	ROS	Restrains ROS level by targeting NOX4		[111]
**p53**	P21	Boosts antioxidant defense		[85]

This table lists several genes, proteins, microRNAs, and lncRNAs, acting as modulators of the ferroptosis process. The molecules are divided according to their pro- or anti-ferroptotic action. The molecular mechanism through which these molecules act is also illustrated.

**Table 2:** Drugs and compounds modulating ferroptosis in cancer cells.

Drugs	Targets	Mechanisms	References
erastin	VDAC2/3 and System *x*_c_^−^	inhibition of the cystine–glutamate antiporter	[28,43]
sorafenib	VEGFR, PDGFR, RAF, GSH, and System *x*_c_^−^	inhibition of the cystine–glutamate antiporter	[126,127,128]
sulfasalazine (SAS)	System *x*_c_^−^	inhibition of the cystine–glutamate antiporter	[129]
cisplatin	GSH	depletion of intracellular GSH	[119]
l-buthionine sulfoximine (BSO)	GCLC	inhibition of GSH synthesis through γ- glutamylcysteine synthetase	[47,130]
artesunate (ART)	lysosomal iron	iron-mediated ROS generation	[6,131,132]
lanperisone	GSH and System *x*_c_^−^	inhibition of cysteine-glutamate antiporter	[132]
RSL3	GPX4	inhibition of GPX4	[79]
altretamine (hexamethylmelamine)	GPX4	inhibition of GPX4	[120]
ML162, DPI compounds	GPX4	GPX4 inactivation and GSH deletion	[133]
FIN56	CoQ10 and GPX4	CoQ10 deletion and GPX4 inactivation	[51]
FINO2	GPX4	GPX4 inactivation and lipid peroxides accumulation	[134]
Statins	HMG	CoQ10 deletion	[135]
trigonelline, brusatol	NRF2	NRF2 inhibition	[86]
siramesine, lapatinib	Ferroportin, TF	increased cellular iron	[136]
BAY 87-2243	ETC	inhibition of mitochondrial respiratory chain	[137]
iron ionophores		sequestration of iron into lysosomes and stimulation of ferritin degradation	[138]
poly(butylcyanoacrylate) and zero-valent iron nanoparticles and arginine-rich manganese silicate nanobubbles		induction of oxidative stress and lipid peroxidation	[139]
Nanocarriers (doxorubicin into mesoporous carbon nanoparticles/withaferin A/poly(ethylene glycol)-coated (PEGylated) silica nanoparticles (C’ dots )		induction of oxidative stress	[122,123,125]

This table summarizes the available chemotherapeutic agents and targeted compounds able to induce or inhibit ferroptosis. Specific targets and mechanisms are also reported.

**Table 3:** Main methods to characterize mitochondrial function in ferroptosis.

Biological Context	Reagents	Functions	References
Morphological changes	TEM	detects ultrastructural mitochondrial morphology changes in the occurrence of ferroptosis	[142]
Mitochondrial oxidative stress	MitoSOX	detects mitochondrial superoxide formation in live cells	[149]
	MitoTEMPO	mitochondrially targeted antioxidant, a specific scavenger of mitochondrial superoxide; it can be used in combination with MitoSOX reagent as positive control	[150]
	Mitotracker	fluorescent dye that stains mitochondria in live cells and its accumulation is dependent upon membrane potential; in can be also used coupled with MitoSOX, in order to stain mitochondrial superoxide and mitochondria together	[26]
Lipid peroxidation	BODIPY	detects reactive oxygen species generated by lipid peroxidation in mitochondrial and plasma membranes using flow cytometry	[151]
ΔΨm	TMRE	quantifies changes in mitochondrial transmembrane potential (ΔΨm) in live cells by flow cytometry, microplate spectrophotometry and fluorescent microscopy	[152]

This table summarizes the available methods and reagents used to explore the pivotal mechanisms and alterations involving mitochondrial function in ferroptosis. Biological context and specific functions are also illustrated for each reported method.

**Table 4:** Mitochondrial markers of ferroptosis.

Classification	Molecule	Mechanisms	References
Energetic metabolism markers	*FH*	its loss of function mutation confers resistance to cysteine-deprivation induced ferroptosis	[26]
	DLD	blocks the increase of L-ROS amount and ΔΨ_m_ caused by cystine deprivation- or sulfasalazine treatment-induced ferroptosis in head and neck cancer	[159]
	GLS1/2	catalyze the conversion of glutamine into glutamate	[26,34]
	TRANSAMINASES	convert glutamate into -KG through the transamination process	[34,158]
	AOA	inhibits cystine deprivation-induced ferroptosis in MEFs	[34,158]
	*GOT1*	its knockdown inhibits CDI ferroptosis in MEFs	[34,158]
	ACSF2	forms an activating thioester bond between the fatty acid and CoA	[34]
	CS	catalyzes the first reaction of the TCA, condensing acetyl-CoA and oxaloacetate to form citrates	[26,34]
Iron metabolism markers	IRON	alterations in (Fe–S) clusters and LIP amount contribute to accumulation of ROS	[14,29]
	Mfrn1/2	iron accumulation and oxidative damage	[30,32,167,188]
	FtMt	protects against the increase of mitochondrial ROS though its storage and ferroxidase activity	[76]
	FLVCR1b	iron export mechanism out of mitochondria	[189]
	ABCB7/8	mitochondrial Fe–S cluster export	[190,191]
	CISD1	regulates mitochondrial iron uptake and generation of mitochondrial lipid peroxides	[29]
Others	VDAC2/3	control the trafficking of ions and metabolites between cytosol and mitochondria, leading an enhanced absorption of mitochondrial iron	[169,170]
	FSP1	mitochondrial effector of apoptotic cell death, able to convert CoQ10 in ubiquinol, that traps lipid peroxyl radicals	[57]
	BID	acts as a connection bridge between surface death receptors and the core apoptotic pathway in mitochondria	[27]
	LONP1	mediates the selective degradation of misfolded or oxidatively damaged polypeptides in the mitochondrial matrix and maintain the integrity of the mitochondrial genome	[187]

This table reports mitochondrial proteins and the relative molecular mechanisms regulating ferroptosis.
